# Disrupted macrophage metabolic adaptation and function drive senescence-induced decline in vertebrate regeneration

**DOI:** 10.7150/thno.111352

**Published:** 2025-06-20

**Authors:** Audrey Barthelaix, Claudia Terraza-Aguirre, Yalén Del Río-Jay, Candice Bohaud, Jérémy Salvador, Marie Morille, Miguel Godinho Ferreira, Christian Jorgensen, Farida Djouad

**Affiliations:** 1 IRMB, Univ Montpellier, INSERM, Montpellier, France.; 2 ICGM, Univ Montpellier, CNRS, ENSCM, Montpellier, France.; 3 Institut universitaire de France (IUF), Paris, France.; 4 Institute for Research on Cancer and Aging of Nice (IRCAN), UMR7284, INSERM U1081, CNRS, Université Cote d'Azur, 06107, Nice, France.; 5 CHU Montpellier, Montpellier, France.

**Keywords:** regeneration, senescence, macrophage, polarization, phagocytosis, metabolism

## Abstract

Rationale: Senescent cells accumulate with age and contribute to impaired tissue regeneration. Here, we developed a senescence-accelerated zebrafish (SAZ) model, characterized by accelerated senescence-like traits and a significant impairment in caudal fin regeneration.

Methods: To investigate the underlying mechanisms of this regenerative defect, we employed a multifaceted approach. We used transgenic zebrafish lines for 4-D tracking of macrophage subsets during regeneration and performed parabiosis to assess the impact of systemic factors. Then, we isolated macrophages by FACS-sorting for a comprehensive transcriptomic study using RT-qPCR, enabling us to analyze both senescence markers and metabolic markers specifically within SAZ macrophages. Furthermore, we conducted phagocytosis assays to evaluate macrophage function. To explore the role of specific metabolic pathways, we used pharmacological treatments with oligomycin and galloflavin.

Results: Our findings revealed that the reduced regenerative potential in SAZ was partly attributable to an impaired macrophage response during regeneration. We observed higher expression of the senescence marker *cdkn2a/b* in SAZ macrophages, which correlated with their reduced ability to polarize into a pro-inflammatory phenotype and exert efficient phagocytosis. These observations were linked to a significant downregulation of *ldha*, a key enzyme in lactate production, specifically within SAZ macrophages at 24 hours post-amputation. Enhancing anaerobic glycolysis in the SAZ model during early regeneration restored *ldha* expression, normalized macrophage activation dynamics, and ultimately rescued caudal fin regeneration. This rescue was entirely abolished by co-treatment with galloflavin, a direct inhibitor of LDH isoforms A and B, thereby underscoring the critical role of lactate metabolism in the regenerative process.

Conclusion: Collectively, our findings demonstrate that senescence impairs regeneration by altering macrophage metabolic adaptation and functions, providing novel insights into the interplay between aging and regenerative capacity.

## Introduction

During ageing, many physiological and biological changes, particularly cellular senescence, metabolic reprogramming, inflammation and matrix changes, may occur, leading to a gradual decline in tissue regeneration 1-3. In humans, the capacity to regenerate damaged or lost tissues diminishes with age and is eventually lost. Conversely, zebrafish retain remarkable regenerative capacities in various tissues, including fins, heart, kidneys and spinal cord, even in old age 4-7. Nevertheless, even in zebrafish, telomere length, telomerase expression and regenerative abilities decrease with age 8-10. Although high *tert* expression levels have been reported in almost all zebrafish tissues at different life stages (larvae, juveniles and adults) 11, *tert* mRNA expression significantly decreases in old fish tissues 8. In the regenerating fins of old fish, the upregulation of telomerase expression is weaker and correlates with the impaired regeneration capacity 8. Consistent with these findings, it was demonstrated on *tert^-/-^* zebrafish that telomere shortening and damage induce cellular senescence, leading to impaired heart regeneration 12. Telomere protection in various eukaryotic organisms requires the shelterin protein complex. Like in humans, the shelterin complex in zebrafish is made of six subunits: TRF1, TRF2, RAP1, TIN2, TPP1 and POT1. TRF1 and TRF2 play a pivotal role by imparting binding affinity and specificity for telomeric double-stranded DNA 10. In zebrafish, TRF2 is encoded by the *terfa* gene that is the ortholog of the human *TERF2* gene. TRF2 protects telomeres by enhancing T-loop formation and inactivating the DNA double strand break kinase ATM and classical nonhomologous end joining at telomeres 13-16. In zebrafish, with age, shelterin gene expression tends to decline 17. Moreover, although *terfa*^+/-^ zebrafish reach adulthood, they exhibit premature ageing 18.

Due to the link between cell senescence, ageing and reduced regeneration potential in regenerative species/models, it may be possible to identify novel therapeutic targets relevant to regenerative medicine by studying the key elements of this association. However, the mechanisms responsible for the regenerative potential decline with age are still not fully understood. For example, short telomeres in the gut lead to inflammation and DNA damage 19 and with age, telomere shortening results in chronic systemic inflammation that contributes to increasing tumor risk 20. Thus, cell-intrinsic factors (senescence mechanisms) and cell-extrinsic factors (changes in the inflammatory microenvironment), which are possibly related, might play a central role in the ontogenetic decline of the regeneration potential 1, 2, 21. Senescent cells establish an inflammation-prone environment reminiscent of ageing, associated with an impairment of regenerative capacity 1, 2. Concerning changes in the inflammatory environment, induced in response to zebrafish caudal fin amputation, we previously showed that transient expression of *tnfa*, coordinated by macrophage subsets, is one of the key signals during the early phases of regeneration 22. Consistent with our findings, it has been shown that although TNF is commonly regarded as pro-inflammatory, it plays a more nuanced role in activated macrophages by selectively modulating their tissue-repair functions rather than broadly suppressing them 23. Thus, the controlled contribution of macrophage-dependent TNF signaling to tissue regeneration shows that well-regulated acute inflammation is crucial for proper regeneration.

Although the permissive role of macrophages in zebrafish larval regeneration has been well characterized by our team 22, 24, the specific impact of cellular senescence on their regenerative functions remains poorly understood. In particular, a critical gap in our knowledge persists regarding how cellular senescence specifically affects these macrophage-dependent regenerative functions. Key questions remain: Do senescent macrophages exhibit impaired polarization or reduced pro-regenerative activity? Does this macrophage dysfunction causally impair regeneration? How does senescence alter macrophage metabolism, and could restoring metabolic activity rescue the regenerative process? Addressing these questions is essential for a complete understanding of macrophage involvement in regeneration.

Cellular senescence and metabolism are linked and many changes in body composition associated with age have their roots in the fundamental processes of ageing. In all organisms, energy production declines progressively with age, mainly as a result of reduced mitochondrial function 25, 26. As regeneration triggers a metabolic adaptation required for cell identity transitions and re-entry into the cell cycle to allow blastema formation and regeneration 27, it is tempting to speculate that senescence-related metabolic changes may alter the metabolic adaptive capacity of the cells present in the injured tissue and their functions essential for regeneration. This hypothesis is supported by studies in skeletal muscle showing that its regenerative potential is tightly related to its metabolism and that age-related metabolic state alterations can directly influence the activity of muscle stem cells (for a review see 28).

To understand why and how the regenerative capacity varies during ontogeny, we developed an approach to assess and compare the regeneration of homologous structures in young and senescence-accelerated zebrafish. We used wild-type (WT) and senescence-accelerated zebrafish (SAZ) larvae to (1) compare their regenerative potential, (2) study the recruitment of functionally distinct macrophage subpopulations to the wound after caudal fin amputation, (3) investigate the role of accelerated senescence on the macrophage response during caudal fin regeneration, and (4) identify the molecular mechanisms that in SAZ larvae, prevent the establishment of a permissive environment for the macrophage response, thereby repressing caudal fin regeneration. We found that SAZ macrophages cannot properly respond to the signals induced upon caudal fin amputation and therefore, cannot provide the correct regenerative environment. Our study reveals a mechanism in which senescence alters macrophage plasticity and regenerative functions, thereby repressing tissue regeneration.

## Results

### *Terfa* silencing does not alter zebrafish larva development

**Figure 1:**
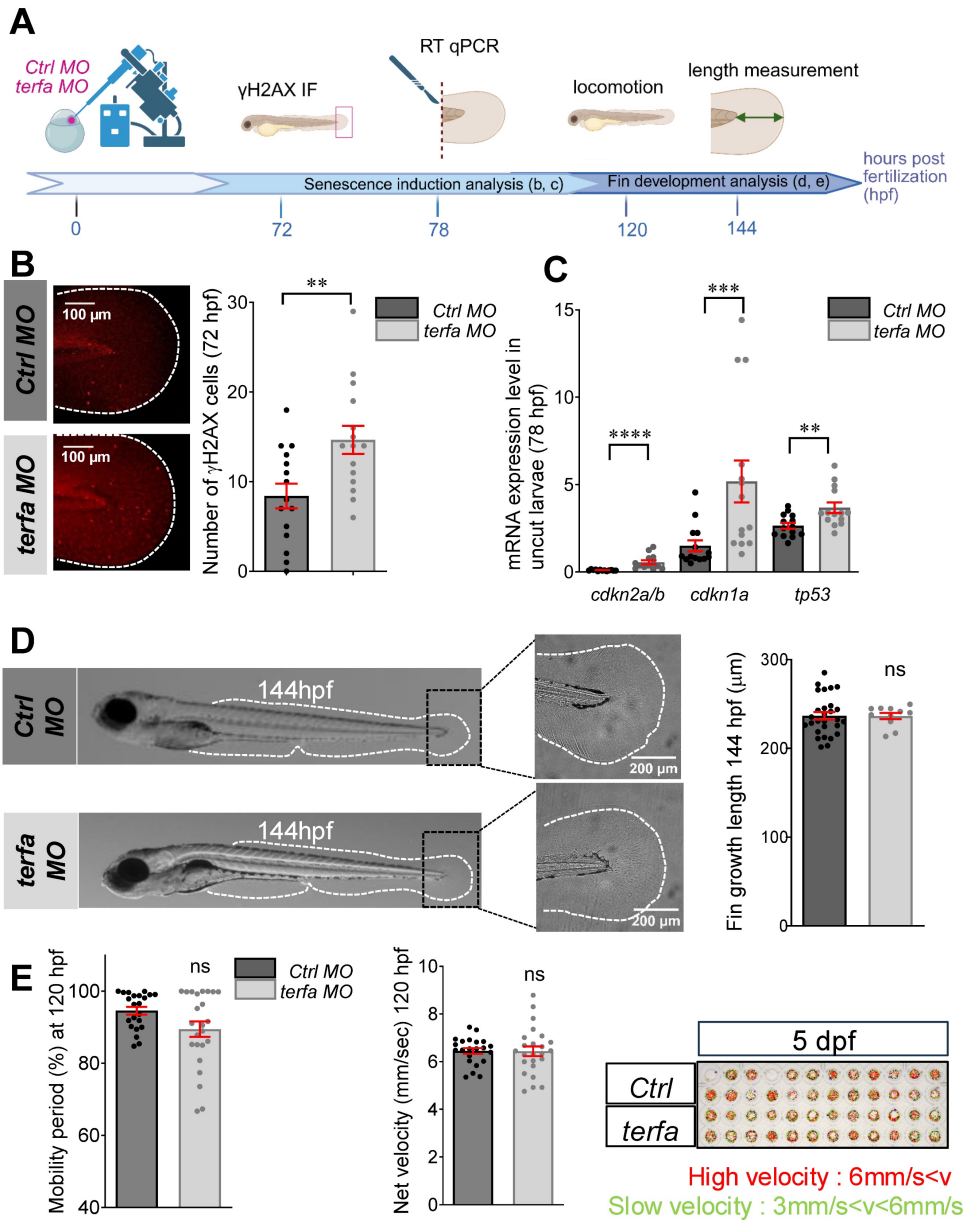
***terfa* knockdown does not affect the development of zebrafish larvae.** (**A**) Experimental design: injection of *terfa* morpholino (*terfa MO*) or control morpholino (*ctrl* MO) at the 1-cell stage. The senescence phenotype was analyzed at 72 h post-fertilization (hpf) by immunofluorescence and at 78 hpf by RT-qPCR. Fin development and function were analyzed by measuring fin length at 144 hpf and zebrafish locomotion and activity at 120 hpf, respectively. (**B**) Confocal images of immunofluorescence staining to assess ɣH2AX expression in the caudal fin of *ctrl* morphants (*ctrl* MO) and *terfa* morphants (*terfa* MO) at 72 hpf. The graph shows the quantification of ɣH2AX-positive cells in the caudal fin; data are the mean ± SEM, n = 15 larvae/group; ** *p* < 0.01 (Mann Whitney test). (**C**) Relative *cdkn2a/b* (p15/16), *cdkn1a* (p21) and *tp53* (p53) expression in the caudal fin of *terfa* MO and *ctrl* MO was assessed by RT-qPCR at 78 hpf. *Ef1a* was used as reference gene and data are the mean ± SEM (n = 14 independent experiments); **** p < 0.0001, *** p < 0.001, ** p < 0.01 (Mann Whitney test). (**D**) Representative images of zebrafish larvae at 144 hpf (left panels) and zoom on the caudal fin folds at 144 hpf (right panels). The graph shows the fin length (mean ± SEM) at 144 hpf in *ctrl* MO (n = 27) and *terfa* MO (n = 11) larvae; ns, not significant (Mann Whitney test). (**E**) Spontaneous locomotion analysis in *ctrl* and *terfa* morphants at 120 hpf. Quantification of the mobility period (left panel) and net velocity (middle panel) during locomotion; n = 24 *ctrl* MO and n = 24 *terfa* MO; ns, not significant (Mann Whitney test). On the right panel, the red and green trajectories correspond to fast and slow swimming, respectively.

To investigate *terfa* role in zebrafish fin fold development and function, we knocked down *terfa* using a morpholino (MO) antisense oligonucleotide against the ATG site (Figure 1A). First, we demonstrated MO-induced blockade of *terfa* translation in developing zebrafish larvae at 3 and 4 days post-fertilization (dpf) by Western blotting (Figure S1A). Then, we assessed the induction of senescence in the fin fold of *terfa* morphants (*terfa* MO) at 72- and 96-hours post-fertilization (hpf) using some markers from the SenNet guidelines to define senescent cells 29. The number of γH2AX-positive cells was increased in *terfa* morphants compared with control morphants (*ctrl* MO) (Figure 1A-B and Figure S1B). *Terfa* knockdown also significantly increased the expression of the cell cycle arrest markers *cdkn2a/b* (p15/16)*, cdkn1a* (p21) and* tp53* (p53) compared with *ctrl* morphants (Figure 1C). Moreover, the expression of interleukin-6 (*il-6*), one of the most prominent cytokines of the SASP 30, was increased in the fin fold of *terfa* morphants compared with *ctrl* morphants as well as the SASP factor *mmp9* (Figure S1C). We followed caudal fin fold development up to 144 hpf by measuring the fin fold length from the end of the notochord to the most proximal end of the fin fold and we did not find any difference between *terfa* morphants and *ctrl* morphants (Figure 1D). We obtained similar results on fin fold development when we knocked down *terfa* using the CRISPR/Cas9 method (terfa CRISPR and *ctrl* CRISPR, respectively) (Figure S1E-F). Lastly, we evaluated the mobility of *terfa* morphants and *ctrl* morphants and *terfa CRISPR and ctrl CRISPR* larvae at 120 hpf using the Zebrabox recording system (see Materials and Methods). We did not observe any effect of *terfa* knockdown on the duration of the mobility period and net velocity (Figure 1E and Fig. S1G). Overall, these results indicate that *terfa* silencing produces zebrafish larvae with a senescent-like phenotype, referred to as senescence-accelerated zebrafish (SAZ) larvae, with normal fin fold development and function.

### *Terfa* silencing impairs caudal fin regeneration

**Figure 2:**
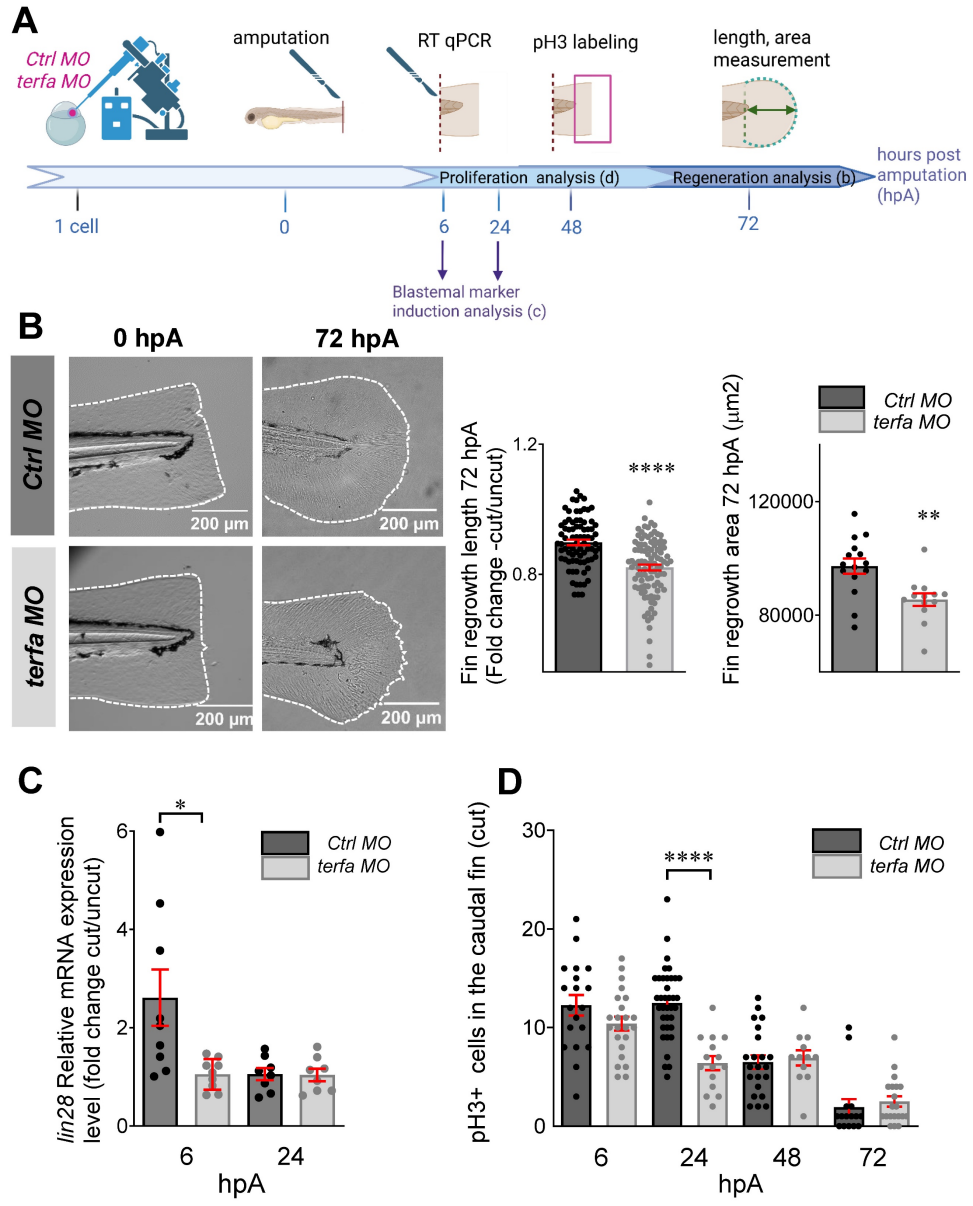
***terfa* knockdown impairs regeneration in zebrafish larvae.** (**A**) Experimental design: injection of *terfa* morpholino (*terfa* MO) or control morpholino (*ctrl* MO) at the 1-cell stage followed by caudal fin section at 72 hpf. Then, *lin28* (blastemal marker) expression in the caudal fin was assessed by RT-qPCR at 6 hpA and 24 hpA. Caudal fin regeneration was monitored by measuring the regenerated caudal fin length and area at 72 hpA and by quantifying cell proliferation by immunofluorescence at 6, 24, 48 and 72 hpA. (**B**) Representative images of the amputated and regenerated caudal fins at 0 and 72 hpA, respectively (left panels). Graphs showing the caudal fin fold length (mean ± SEM) in* ctrl* and* terfa* morphants at 72 hpA (middle panel) and the fin area (mean ± SEM) in* Ctrl* and* terfa* morphants at 72 hpA (right panel); **** p < 0.0001, ** p < 0.01 (Mann Whitney test) (**C**) Relative *lin28* expression in the caudal fin of *ctrl* and* terfa* morphants assessed by RT-qPCR at 6 and 24 hpA; *ef1a* was used as reference gene (data are the mean ± SEM, n = 8 and n = 9 independent experiments at 6 and 24 hpA, respectively); * *p* < 0.1 (Mann**-**Whitney test). (**D**) Quantification of cell proliferation in the regenerated caudal fin fold by assessing pH3 expression at 6, 24, 48 and 72 hpA; **** p < 0.0001 (Mann**-**Whitney test).

We assessed the effect of early senescence, induced by *terfa* knockdown, on the regeneration potential. First, we demonstrated MO-induced blockade of *terfa* translation in regenerating zebrafish larvae at 0 and 24 hpA by Western blotting (Figure S2A). Then, at 72 hpf, we amputated the caudal fin fold of *terfa* morphants and *ctrl* morphants (MO injected at the 1-cell stage) and monitored their regeneration (Figure 2A-B and Figure S2B). At 6 hpA (78 hpf), expression of the cell cycle arrest markers *cdkn2a/b*, *cdkn1a* and *tp53* was significantly higher in the amputated area of *terfa* morphants than *ctrl* morphants (Figure S2C)*.* Measurement of the regrowth length and surface area of the regenerating caudal fin fold between the notochord and fin tip at 24, 48 and 72 hpA showed that caudal fin length and surface area were significantly smaller in the SAZ than in *ctrl* morphants (Figure 2B and Figure S2B). We obtained similar results when *terfa* was knocked down with the CRISPR/Cas9 approach (Figure S2D-E)*.* We previously showed that regeneration following amputation of an appendage, such as the caudal fin fold in larval zebrafish, involves a stage of blastemal cell proliferation from 6 hpA (78 hpf) in the region close to the stump followed by the spread of cell proliferation to more proximal regions from 24 hpA (96 hpf) before returning to normal levels at 72 hpA (144 hpf) 22. To determine whether a blastema was formed, we assessed by RT-qPCR the expression of *lin28,* a blastema marker 31, 32. *Lin28* expression level was significantly lower in *terfa* morphants than in *ctrl* morphants at 6 hpA (Figure 2C). We then assessed blastemal cell proliferation during regeneration by monitoring the expression of phosphorylated histone 3 (pH3) that marks proliferative cells. The number of pH3-positive cells was similar in *ctrl* morphants and *terfa* morphants at 6 hpA, but became significantly lower in the *terfa* morphant blastema at 24 hpA (Figure 2D). These results suggest that SAZ larvae resulting from *terfa* inactivation display normal caudal fin development, but compromised regenerative capabilities.

### Accelerated senescence impairs regeneration by altering the function of circulating macrophages

**Figure 3:**
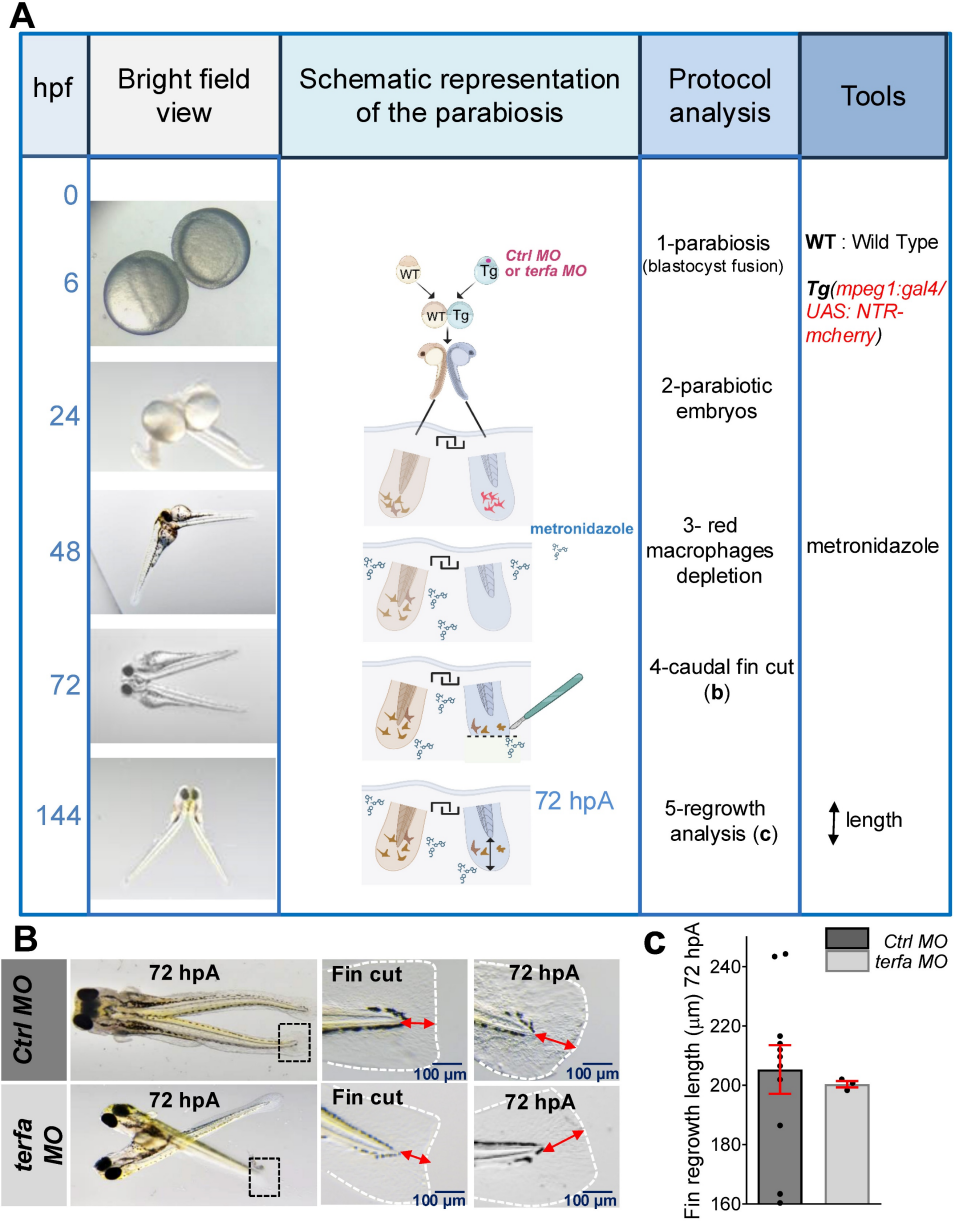
** Wild-type circulating cells partially restore the regeneration potential in *terfa* morphants.** (**A**) Schematic representation of parabiosis experiment using *Tg(mpeg1:gal4/UAS:NTR-mcherry)* larvae previously injected with *terfa* MO or *ctrl* MO and wild-type (WT) larvae. Conjoined embryos were generated at the shied stage. Macrophages in the morphant partner were depleted by injection of 10 mM metronidazole at 48hpf. The morphants' caudal fin folds were amputated at 72 hpf. Then, at 72 hpA, regeneration was analyzed by measuring the length of the regenerated caudal fin fold. (**B**) Representative images of conjoined embryos at 72 hpA. The middle panels show a zoom of the cut caudal fins. The right panels show a zoom of regenerated fins at 72 hpA. (**C**) Quantification of the regenerated caudal fin length at 72 hpA (data are the mean ± SEM, n = 11 *Ctrl* MO larvae, n = 3 *terfa* MO larvae).

Age-related changes in circulating factors can affect tissue homeostasis 33. Therefore, to determine whether a SAZ circulating factor/cell population was implicated in the loss of fin regeneration potential, we surgically generated conjoined zebrafish embryos (one *terfa* morphant and one WT individual) that shared a common circulatory system (parabiosis) 22, 34. We focused on circulating macrophages because we previously showed that macrophage depletion represses the caudal fin fold regeneration potential of zebrafish larvae 22. Thus, to determine whether *terfa-*deficient macrophages were involved in the reduced regeneration potential of *terfa* MO larvae, we performed parabiosis experiments fusing WT embryos and *Tg(mpeg1:gal4/UAS:NTR-mCherry)* embryos in which the *terfa* MO or *ctrl* MO had been injected (Figure 3A and Figure S3A). In the resulting MO-injected *Tg(mpeg1:gal4/UAS:NTR-mCherry)* zebrafish line, we depleted macrophages by addition of 10 mM metronidazole (MTZ) at 48 hpf, and then we amputated the caudal fin of the *terfa* morphant or *ctrl* morphant parabionts at 72 hpf. At 72 hpA, caudal fin fold regeneration was comparable in the *terfa* morphant parabiont and* ctrl* morphant parabiont, indicating that the recruitment of WT macrophages at the wound site of the amputated *terfa* morphant parabiont rescued its regeneration potential (Figure 3B-C and Figure S3A). Overall, these findings suggest that *terfa*-deficient senescent macrophages are implicated in the impaired caudal fin regeneration in SAZ larvae.

### *Terfa* silencing in zebrafish larvae increases the frequency of senescent macrophages with reduced activation potential in the regenerating caudal fin

**Figure 4:**
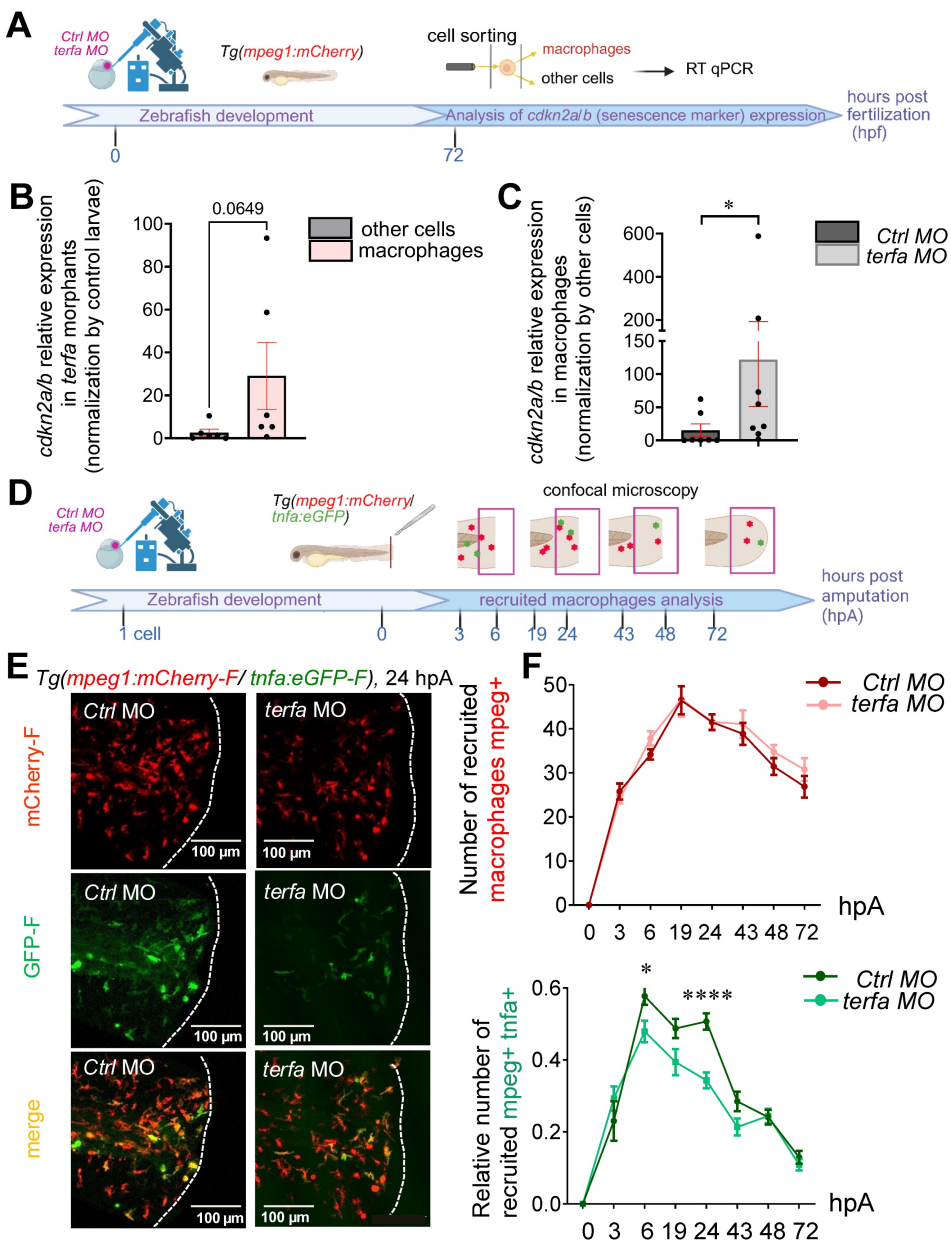
***terfa* knockdown impairs the macrophage response following caudal fin amputation.** (**A**) Experimental design: injection of *terfa* morpholino (*terfa* MO) or control morpholino (c*trl* MO) at the 1-cell stage in the zebrafish transgenic line *Tg*(*mpeg1:mCherry*). Macrophages were isolated from both *ctrl* morphants and *terfa* morphants (100 larvae per condition) at 72hpf. The *p15/16* expression level was measured and compared between the sorted macrophages and other cells populations (other cells), consisting of all non-macrophage cells from the zebrafish larva using qRT-PCR. (**B**) Relative mRNA expression levels of *cdkn2a/b* in the *terfa* morphants were assessed at 72 hpf by qRT-PCR. *Ef1a* was used as reference gene (data are the mean ± SEM, n = 6 independent experiments, Mann**-**Whitney tests were performed, *p = 0.0649*). (**C**) Relative mRNA expression level of *cdkn2a/b* in macrophages at 72hpf. *Ef1a* was used as reference gene (data are the mean ± SEM). * p < 0.1 (**D**) Experimental design: injection of *terfa* morpholino (t*erfa* MO) or control morpholino (*ctrl* MO) at the 1-cell stage in *Tg(mpeg1:mcherry-F/tnfa:eGFP-F)* larvae. Caudal fins were amputated at 72 hpf and macrophage recruitment was analyzed from 3 to 72 hpA. (**E**) Representative images of maximal projections of caudal fin of *Tg(mpeg1:mcherry-F/tnfa:eGFP-F) ctrl* or *terfa* morphants at 24 hpA. All macrophages (mCherry-labeled) are in red and *tnfa+* macrophages are in green. (**F**) Total number of macrophages (red) recruited to the wound area in *ctrl* (dark red) and *terfa* (pink) morphants at the indicated time points after amputation over 3 days (upper panel). Relative number of *tnfa+* macrophages among all macrophages at the wound site in *ctrl* (dark green) and* terfa* (light green) morphants at different time points over 3 days. Data are the mean ± SEM, n = 50 larvae at 6, 24 and 48 hpA, n = 20 larvae at 19, 43 and 72 hpA, and n = 10 larvae at 3 hpA; **** p < 0.0001, * p < 0.1 (Tukey's multiple comparisons test).

To determine whether *terfa*-deficient senescent macrophages contribute to the loss of regenerative potential in SAZ larvae, we first examined whether these macrophages exhibit a senescent phenotype compared with those in *ctrl* morphants, focusing on *cdkn2a/b*, which was overexpressed in *terfa* morphants compared with controls (Figure 1C). By comparing *cdkn2a/b* expression levels in sorted macrophages versus all other non-macrophage cells from zebrafish larvae (Figure 4A), we found that *cdkn2a/b* expression levels tended to be higher in macrophages than in other cells in *terfa* morphants (Figure 4B). Moreover, *cdkn2a/b* expression levels were significantly higher in macrophages isolated from *terfa* morphants than *ctrl* morphants (Figure 4C). Caudal fin amputation in zebrafish larvae induces the recruitment of macrophages to the site of injury and their activation and polarization 22, 24. To determine whether an alteration of the functions of *cdkn2a/b*-overexpressing *terfa*-deficient senescent macrophages could be involved in the loss of fin regeneration potential in SAZ larvae, we focused on macrophage migration and activation because they are essential for regeneration. First, we determined the recruitment kinetics of macrophage subsets after caudal fin amputation in zebrafish at 72 hpf. To trace macrophage subsets, we used *Tg(mpeg1:mCherry-F/tnfa:eGFP-F)* zebrafish larvae, in which macrophages positive for *tnfa*, a pro-inflammatory cytokine marker of macrophages, express both farnesylated eGFP (GFP-F) and mCherry (mCherry-F) 24 (Figure 4D). After knocking down *terfa* in these zebrafish larvae, we imaged them at different time points after caudal fin amputation. Consistent with our previous studies 22, 24, in both *terfa* morphants and *ctrl* morphants, *mpeg1*+ macrophages (all macrophages labeled by red fluorescence) were recruited rapidly to the wound site and remained present until complete fin regeneration at 72 hpA (Figure 4E-F). Moreover, in accordance with our previous studies, *tnfa*+ macrophages (green) mainly accumulated at the wound site from 6 hpA and up to 24 hpA in both *terfa* morphants and *ctrl* morphants (Figure 4E). However, the number of *tnfa*+ macrophages from 6 to 24 hpA was lower in *terfa* morphants than *ctrl* morphants (Figure 4F). At these post-amputation times, the *lin28* expression level and the number of proliferative pH3-positive cells and were significantly lower in *terfa* morphants than *ctrl* morphants (Figure 2B-C). This suggests that the impaired caudal fin regeneration potential in SAZ may be due to lower *tnfa* production by senescent pro-inflammatory macrophages.

### Oligomycin reverses the impaired macrophage response and rescues regeneration in *terfa* morphants

**Figure 5:**
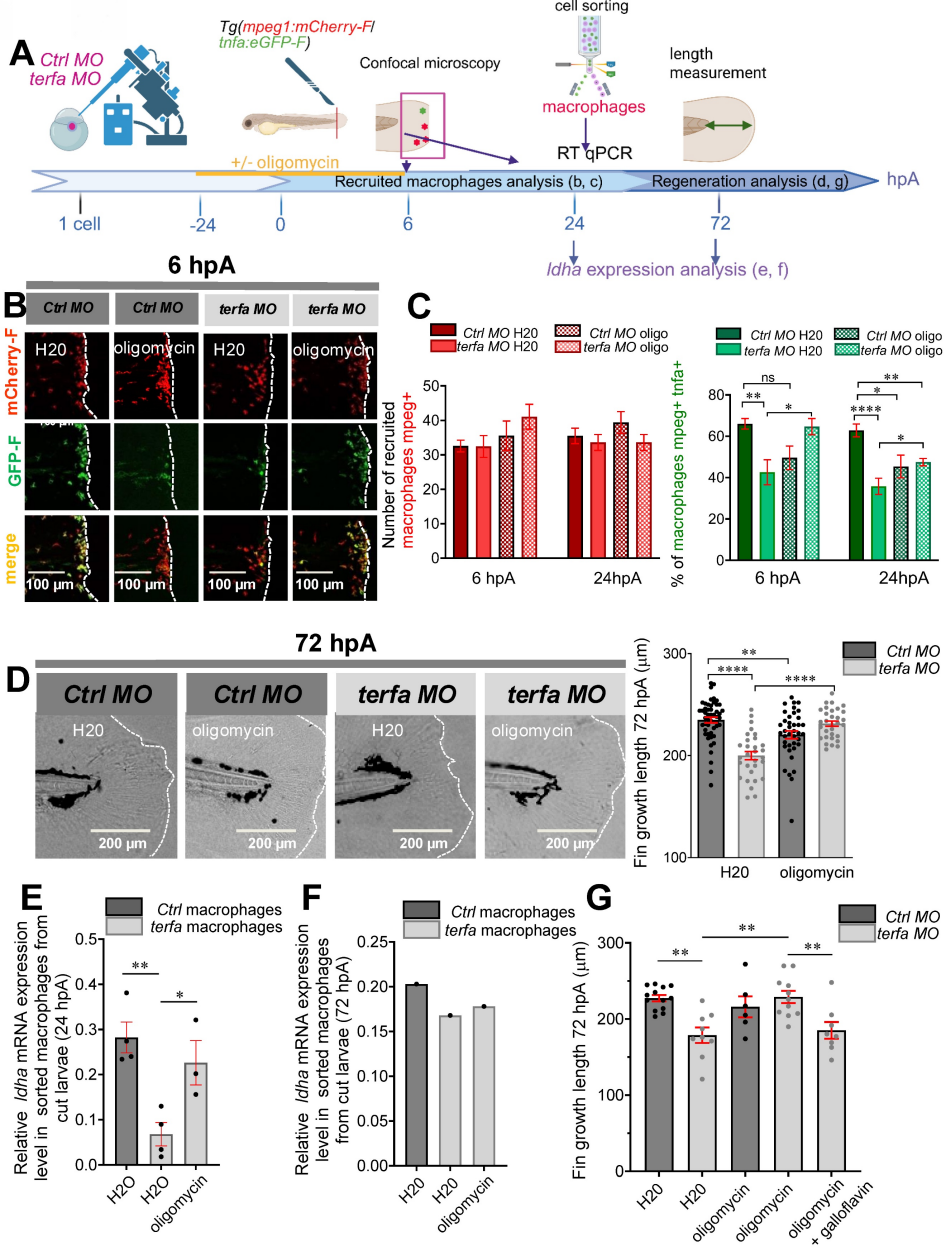
** Oligomycin restores the macrophage response and regenerative potential in *terfa* morphants.** (**A**) Experimental design: injection of *terfa* morpholino (*terfa* MO) or control morpholino (*ctrl* MO) at the 1-cell stage in the transgenic line *Tg*(*mpeg1:mcherry-F; tnfa:eGFP-F*). Amputation of the caudal fin fold at 72 hpf (corresponding to 0 hpA in the workflow). Oligomycin was added to the fish water 24 hours before amputation and maintained in the medium until 6 hours post-amputation (6 hpA). The recruitment of macrophages was assessed at 6 and 24 hpA. Macrophage cell sorting was performed at 24 hpA and *ldha* expression in macrophages isolated from *ctrl* larvae and *terfa* morphants was analyzed by RT-qPCR at 24 hpA and 72 hpA. Finally, fin regrowth was measured at 72 hpA. (**B**) Representative images of confocal maximal projections of *Tg(mpeg1:mcherry-F/tnfa:eGFP-F) ctrl* or *terfa* morphants' caudal fin at 6 hpA. Zebrafish larvae were incubated or not (H_2_O) with oligomycin (oligo) from 48 hpf to 6 hpA. (**C**) In red, number of *mpeg+* macrophages recruited at the wound site in treated (oligo) and untreated (H_2_O) *ctrl* MO (dark red) and *terfa* MO (light red) larvae at 6 and 24 hpA (upper panel). In green, percentage of *tnfa+* macrophages recruited to the wound site in treated (oligo) and untreated (H_2_O) *ctrl* MO (dark green) and *terfa* MO (light green) larvae at 6 and 24 hpA. Data are the mean ± SEM, 10 < *n < 2*0 larvae for each condition; **** p < 0.0001, ** p < 0.01 and * p < 0.1 (Tukey's multiple comparisons test). (**D**) Bright-field images of the regenerating caudal fin of treated (oligomycin) and untreated (H_2_O) *terfa* or *ctrl* morphants at 72 hpA. The graph shows the fin regrowth length (mean ± SEM); 30 < *n* < 51 larvae per condition; **** p < 0.0001, ** p < 0.01 (1 way ANOVA, Tukey test, with multiple comparisons). (**E**) Relative expression level of *ldha* (relative to *ef1a*) in sorted macrophages of treated (oligomycin) and untreated (H_2_O) *terfa* or *ctrl* morphants at 24 hpA (data are the mean ± SEM, 3 < *n* < 4 independent experiments ** p < 0.01 * p < 0.01 (1 way ANOVA, Tukey test, with multiple comparisons). (**F**) Relative expression level of *ldha* (relative to *ef1a*) in sorted macrophages of treated (oligomycin) and untreated (H20) *terfa* or *ctrl* morphants at 72 hpA (one experiment). (**G**) The graph shows the fin regrowth length (mean ± SEM) at 72 hpA, *ctrl* morphants and *terfa* morphants were incubated either in water (H₂O), in oligomycin, or in oligomycin combined with galloflavin. ** p < 0.01 (1 way ANOVA, Tukey test, with multiple comparisons).

As macrophage functions are partly governed by their metabolic status (for review see 35), we wondered whether in SAZ larvae, accelerated ageing altered macrophage functions in the regenerating caudal fin by affecting their metabolism. For instance, glycolysis inhibition affects many macrophage functions, particularly their ability to secrete pro-inflammatory cytokines. Therefore, glycolysis is a crucial metabolic event for pro-inflammatory macrophage activation (for review see 36) and possibly blastema formation. To determine whether the lower number of *tnfa+* pro-inflammatory macrophages at 24 hpA in *terfa* morphants compared with *ctrl* morphants (Figure 4F) was due to a glycolytic metabolism defect, we promoted glycolysis by treating zebrafish larvae with oligomycin 37 (Figure 5A). As we found that caudal fin regeneration relies on the early and transient accumulation of *tnfa*+ pro-inflammatory macrophages 22, we compared an early and transient enhancement of glycolysis (i.e. oligomycin addition for 30 hours: from 24 hours before to 6 hours after caudal fin amputation) (Figure 5 and Figure S4A-B) and a persistent one (oligomycin for the entire regeneration duration) (Figure S4C). The overall kinetics of macrophage recruitment (*mpeg*+ cells) during the entire regeneration process (from 0 to 72 hpA) was not different in *terfa* morphants and *ctrl* morphants treated with oligomycin (Figure 5B-C and Figure S4A). This suggests that oligomycin does not affect macrophage migration to the wound site in *terfa* morphants and *ctrl* morphants.

Conversely, the significant decrease in the number of *tnfa*+ macrophages at the wound site in *terfa* morphants at 6 and 24 hpA (Figure 5B-C) was reversed by the short oligomycin treatment (Figure 5C). Of note, the number of *tnfa*+ macrophages was significantly decreased at 24 hpA in the regenerating caudal fin of treated *ctrl* morphants (*ctrl* MO Oligo) compared with untreated *ctrl* MO larvae (*ctrl* MO H_2_O) (Figure 5C). Moreover, in treated *terfa* morphants, the number of *tnfa*+ macrophages recruited to the wound site was increased during the first 3 hpA compared with untreated *terfa* morphants (Figure S4B). This result suggests that in *terfa* morphants, the short oligomycin treatment rescued the early inflammatory phase (6 hpA) of the regeneration process required for TNFα production by *tnfa+* macrophages 22. This was followed by a rapid resolution of inflammation, illustrated by the decrease of *tnfa+* macrophages at 24 hpA (Figure 5C and Figure S4B).

As we previously showed that specialized pro-resolving lipid mediators (neuroprotectin/protectin D1) improve fin fold regeneration by accelerating the resolution of inflammation 38, we asked whether oligomycin could rescue the impaired regenerative potential of SAZ larvae. Indeed, the short oligomycin treatment significantly increased fin fold growth in *terfa* morphants (length or/and area from the initial amputation position to the new distal fin fold edge at 72 hpA). Conversely, the long oligomycin treatment similarly reduced regeneration in both *ctrl* and *terfa* morphants (Figure S4C and Figure S4D). In line with the significant decrease in the number of *tnfa*+ macrophages at 6 and 24 hpA in treated *ctrl* morphants (*ctrl* MO Oligo) compared with untreated *ctrl* morphants (*ctrl* MO H_2_O), fin fold growth was significantly impaired at 72 hpA in the *ctrl* MO Oligo group (Figure 5D). This result is consistent with our previous study demonstrating that TNFα-positive early-recruited macrophages are essential for blastema formation and fin-fold regeneration at 72 hpA.

Then, we investigated whether the reduced number of *tnfa⁺* macrophages at the wound site in *terfa* morphants at 6 and 24 hpA (Figure 5B-C), a phenotype restored by short-term oligomycin treatment, was linked to metabolic alterations in macrophages. First, we analyzed the expression of key metabolism-related genes in macrophages isolated from *ctrl* morphants and *terfa* morphants at 24 and 48 hpA. We focused specifically on *ldha, eno3*, and *pdhb*, we previously shown to be selectively upregulated in macrophages during the regenerative process 39. At 24 hpA, a time point when pro-inflammatory macrophages reach a peak before rapidly declining 22, the expression of *ldha*, encoding lactate dehydrogenase A, a key enzyme in pyruvate-to-lactate conversion, was significantly reduced in macrophages isolated from *terfa* morphants compared to *ctrl* morphants (Figure 5E). In the study in which we identified *ldha* as upregulated in macrophages during caudal fin regeneration through single-cell RNA sequencing 39, we also demonstrated that lactate was a key metabolite involved in the early polarization of macrophages toward a pro-inflammatory phenotype. Thus, to determine whether *terfa* silencing specifically affects pro-inflammatory macrophages in a lactate-dependent manner, we further assessed *ldha* expression at 72 hpA (Figure S4E), a time point when pro-inflammatory macrophages are no longer present in the regenerating fin (Figure 5F). At this later stage, *ldha* expression was comparable between *ctrl* morphants and *terfa* morphants, suggesting that *terfa* silencing selectively impairs lactate metabolism in early pro-inflammatory macrophages, which are essential for initiating regeneration. In contrast, the expression of other glycolysis-associated genes, such as *eno3* and *pdhb*, upregulated in macrophages during regeneration 39, was similar in *ctrl* morphants and *terfa* morphants at 24 hpA (Figure S4F-G). We then assessed the effect of oligomycin treatment at 24 hpA and observed that *ldha* expression in *terfa* morphants was restored to levels comparable to those in *ctrl* MO correlating with the recovery of regenerative capacity in *terfa* morphants (Figure 5D-E). Of note, *eno3* and *pdhb* expression remained unchanged (Figure S4F-G). Importantly, the oligomycin-induced restoration of regeneration was completely abrogated by co-treatment with galloflavin (Figure 5G), a direct inhibitor of lactate dehydrogenase isoforms A and B (LDHA/B), which blocks the conversion of pyruvate to lactate. These results indicate that *terfa* deficiency impairs regeneration by altering *ldha* expression in early-phase macrophages, with *terfa* inhibition disrupting macrophage metabolism, an effect further supported by galloflavin-mediated LDHA inhibition, which prevents oligomycin-induced regeneration rescue in the SAZ.

### *Terfa* silencing impairs macrophage phagocytic activity in the regenerating caudal fin

**Figure 6:**
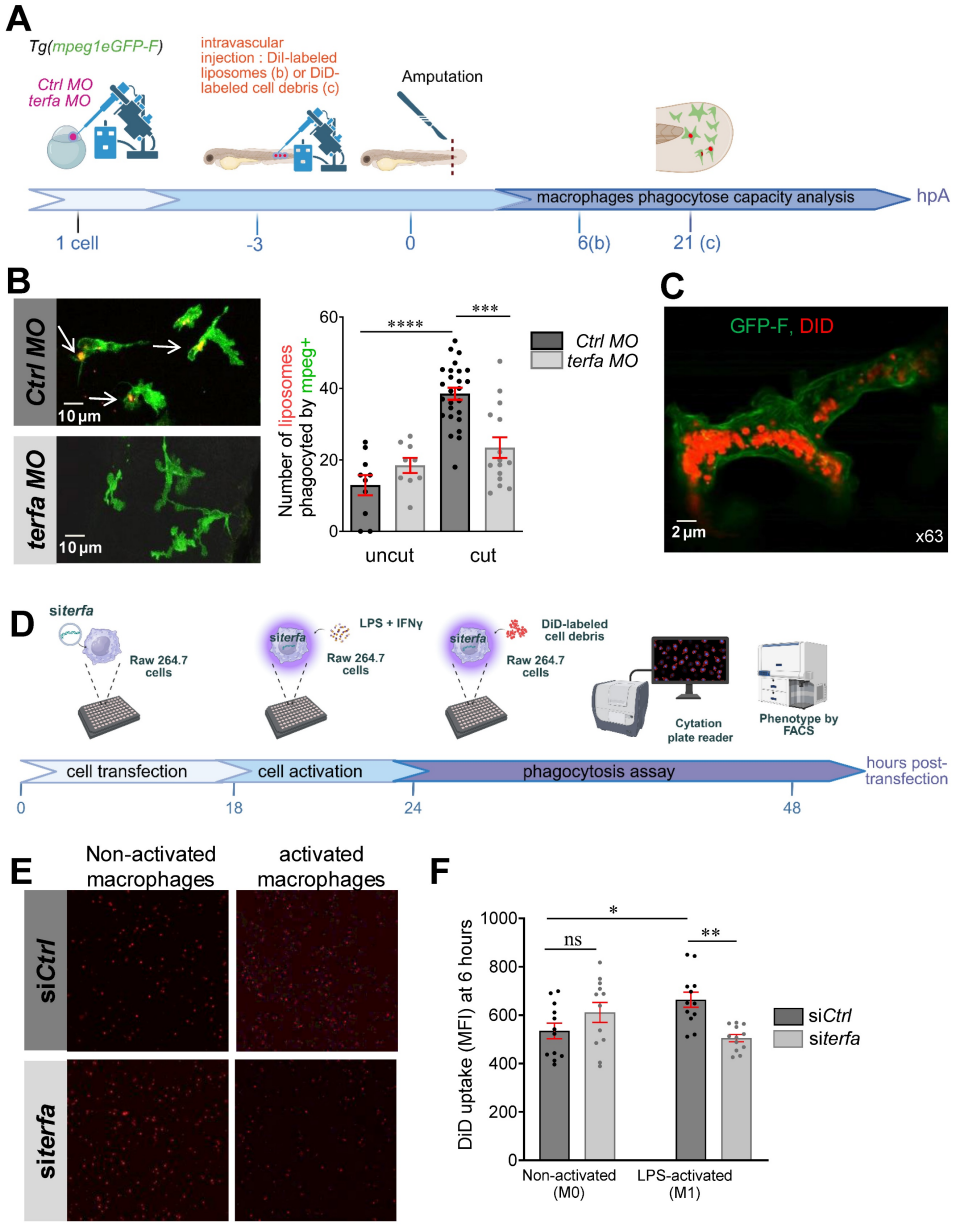
***terfa* knockdown impairs the phagocytic potential of zebrafish and mouse macrophages.** (**A**) Experimental design: injection of *terfa* morpholino (*terfa* MO) or control morpholino (*ctrl* MO) at the 1-cell stage. Injection of DiI-labeled liposomes (or DiD-labeled cell debris) 3 hours before caudal fin amputation. Analysis of macrophage phagocytic activity by confocal microscopy at 6 hpA (for the DiD-labeled cell debris) and at 24 hpA (for DiI-labeled liposomes). (**B**) Confocal maximum projections of the fluorescence signal of DiI-labeled liposomes (red) and GFP-labeled macrophages (green) in *Tg(mpeg1:eGFP) terfa* and *ctrl* morphants at 24 hpA. White arrows indicate liposomes phagocytosed by macrophages (left panels). The graph shows the quantification of phagocytosed liposomes in the caudal fin (mean ± SEM, n = 10 larvae for both uncut group, n = 26 larvae for the amputated *ctrl* MO group and n = 15 larvae for the amputated *terfa* MO group) (right panel); **** p < 0.0001, *** *p* < 0.001 (Kruskal-Wallis test). (**C**) Confocal maximum projection analysis of DiD*-*labeled cell debris (red) fluorescence in one GFP-labeled macrophage (green). (**D**) Mouse RAW 264.7 macrophages (28,000 cells per well) were seeded in a multi-well plate. After 6 hours, cells were transfected with 20 nM of siRNA targeting *terfa* for 18 hours. Then, cells were activated (M1) or not (M0) with LPS (250 ng/mL) + IFN*γ* (20 ng/mL) for 6 hours, followed by DiD-labeled cell debris addition. Cell debris uptake was assessed using the Cytation 5 plate reader for 24 hours. (**E**) Representative images showing the fluorescence intensity indicative of macrophages that have phagocytosed DiD-labeled debris at 6 hours after debris addition. (**F**) Quantitative analysis of fluorescence intensity. Data are the mean fluorescence intensity ± SEM at 6 hours; n=3; **p<0.01, *p<0.1 (1 way ANOVA, Tukey test, with multiple comparisons).

Macrophages play a key role in regeneration also via their phagocytic activity 40, but show a significantly reduced capacity to uptake fluorescent myelin when they come from aged mice compared from neonatal mice or young adults (for a review, see 41). To assess the phagocytic activity of macrophages in *Tg*(*mpeg1:eGFP-F*) larvae (transgenic line in which macrophages are labelled with GFP-F), we injected DiI-labeled liposomes (or DiD-labeled cell debris) at 72 hpf and amputated the caudal fin fold to induce macrophage recruitment and activation at the wound site (Figure 6A). Quantification of the phagocytic capacity by confocal microscopy at 6 and 24 hpA showed that after injury, the number of *mpeg1*-positive macrophages (green) that could engulf DiI-labeled liposomes increased significantly in *ctrl* morphants but not in *terfa* morphants (Figure 6B). Monitoring of macrophage interactions with DiD-labeled cell debris showed the internalization of the labeled debris by macrophages at 6 hpA (Figure 6C and Figure S5A-B and Video 1). Altogether, these results show that the *terfa* morphant macrophage phagocytic capacity is impaired.

To determine whether the macrophage phagocytic activity was altered directly by *terfa* knockdown, we silenced *terfa* using siRNAs in RAW 264.7 murine macrophages (Figure 6D). Transfection of RAW 264.7 macrophages with 50 nM of siRNA against *terfa* significantly decreased *terfa* expression by more than 50% compared with control cells (control siRNA) (Figure S5C). Then, at 18 hours post-transfection, we activated RAW 264.7 macrophages with lipopolysaccharide (LPS; 250 ng/mL) and interferon (IFN)-g (20 ng/mL) for 24 hours before addition of DiD-labeled cell debris (from mesenchymal stromal cell lysates). DiD-labeled cell debris uptake quantification for 48 hours showed that it was higher in activated than non-activated control macrophages (Figure 6E-F). Conversely, it did not increase in activated *terfa*-deficient macrophages. These data show that *terfa* knockdown alters the phagocytic capacity of activated macrophages, irrespective of the tissue or species of origin.

## Discussion

In this study, we showed that caudal fin regeneration is impaired in SAZ larvae, which display accelerated senescence-like characteristics. This decline is linked to significant changes in macrophage functions that are essential for regeneration, particularly their polarization and phagocytic capacities. This macrophage-dependent decline in zebrafish regenerative capacity can be reversed by transiently promoting glycolysis at the expense of mitochondrial oxidation during the initial phases of regeneration.

Our study provides an effective approach for inducing senescence in zebrafish. Indeed, compared with controls, *terfa* knockdown in zebrafish larvae significantly increased (i) the number of γH2AX-positive cells, (ii) the expression of the cell cycle arrest markers *cdkn2a/b, cdkn1a* and* tp53*, and (iii) the presence of SASP components, such as IL-6 and MMP9. Moreover, while the macroscopic development of the caudal fin fold was not affected in SAZ larvae up to 144 hpf, cell proliferation in the caudal fin was decreased at 96 hpf and was then compensated at 120 and 144 hpf. This may be because MOs affect all embryonic cells, but their effects persist only for 5-6 days—matching the timeframe of our study, which examined regeneration during the first 6 days post-fertilization. Therefore, the impact of *terfa* knockdown on cell proliferation in the caudal fin fold at 96 hpf, followed by compensation at 120 and 144 hpf (i.e. day 5 and 6 post-fertilization), resulted in no effect on caudal fin morphology at 144 hpf.

Second, in the context of tissue regeneration, we showed that *terfa* knockdown induced a significant increase in cell cycle arrest markers (*cdkn2a/b, cdkn1a* and* tp53*) after caudal fin amputation that was associated with a reduction in the regenerative potential of SAZ larvae. This reduction in regeneration potential is consistent with the significantly lower cell proliferation and *lin28* expression in the blastema of SAZ larvae that were not compensated up to day 6. Our results are in line with a previous study showing that 12 days after amputation of part of the caudal fin, aged zebrafish regenerate tissue at a 50% lower rate than younger zebrafish 8.

In mitotic neural cells, the expression of a dominant-negative TRF2 variant leads to the induction of P53 and P21, resulting in senescence 42. Similarly, here we found that *terfa* knockdown increased the expression of the tumor suppressor gene *tp53*, which is not just a downstream effector of telomere damage signaling, but also one of the main inducers of senescence. Similarly, *cdkn1a*, a cyclin-dependent kinases (CDK) inhibitor negatively regulated by TRF2 43, was significantly increased in response to *terfa* knockdown in both intact and amputated zebrafish larvae. Upon binding to CDKs, P21 represses cell proliferation directly 44 and it was proposed to be sufficient to induce cell senescence 45. Mice overexpressing P21 show signs of muscle pathology, including atrophy, fibrosis and impaired physical function. Here, we observed that *cdkn1a* upregulation in the SAZ occurred concomitantly with a significant reduction in cell proliferation rate in both intact and amputated zebrafish, although no causal relationship was established. This was associated with the reduced regenerative potential of the SAZ caudal fin fold that depends on the formation of the blastema, a highly proliferative structure. This inverse relationship between *cdkn1a* expression level and regeneration potential was described in the super-healing MRL mouse strain 46, 47. Indeed, in this strain, unique among mammals for its ability to regenerate tissues such as ear, heart and cartilage 46, P21 expression is reduced in both uninjured and injured tissues. P21 plays a very important role in tissue regeneration because its deletion alone establishes the ability of appendages to regenerate in whole animals 48. In addition, P53 activity undergoes fluctuations during regeneration. Although P53 inhibition has been documented during the early stages of proliferation, it increases during the final phase of the regeneration process, when maintenance of tissue integrity and fidelity is crucial 49. In SAZ, *tp53* expression level was significantly increased at the early stage of regeneration (6 hpA). The high expression levels of *cdkn1a* and *tp53* induced by *terfa* knockdown in zebrafish might partly explain the impaired regenerative potential. Regarding *cdkn2a/b* expression, we observed a significant increase in *terfa* morphants compared to *ctrl* morphants. Upon closer examination, this senescence marker appeared to be more prominently expressed in macrophages isolated from *terfa* morphants. A crucial consideration when interpreting senescence markers in immune cells, particularly macrophages, is their inherently high expression of canonical senescence markers such as p16INK4a and p21 50-52. Indeed, there is a significant overlap in features between senescent cells and macrophages, including the expression of these markers and even SA-β-galactosidase 50. Their expression in macrophages is well-documented and can be part of their normal physiological responses or inflammatory states, not solely indicative of permanent senescence. However, here, our study focused on the comparative expression profile of several canonical senescence markers between *terfa* morphants and *ctrl* morphants. We observed a clear and significant difference in the expression of *cdkn2a/b* (p15/16) marker between macrophages in *ctrl* morphants and those in *terfa* morphants. This differential expression, despite the high baseline expression in macrophages, highlights a unique biological change in the *terfa* context that warrants further investigation. Therefore, rather than relying on the presence of these markers, our findings emphasize the importance of analyzing their comparative expression levels in distinct cell types. Further investigations are necessary to confirm the hypothetical role of *cdkn1a* and *tp53* in limiting the regenerative capacity of zebrafish following early senescence induced by *terfa* knockdown. In addition, future studies should explore the bidirectional interactions between senescent cells and macrophages—specifically (i) how senescent cells, through their Senescence-Associated Secretory Phenotype (SASP), may recruit and modulate macrophage functions 53, and (ii) how macrophages, in turn, contribute to the regulation or clearance of senescent cells 54, 55.

Age-related changes are known to induce metabolic reprogramming across various cell types within a tissue, potentially compromising its regenerative capacity 56. Among these cells, macrophages play a particularly central role in skin wound healing, where their function is tightly linked to dynamic shifts in metabolic states 57-60. In the early stages of healing, pro-inflammatory macrophages primarily depend on glycolysis to perform tasks such as debris clearance and pathogen elimination. As the process advances, macrophages transition toward an anti-inflammatory phenotype, characterized by a metabolic shift involving lipid and amino acid pathways. While the metabolic plasticity of macrophages is increasingly acknowledged, it remains incompletely understood. In this context, we investigated whether modulating the metabolic microenvironment in SAZ larvae could enhance their regenerative ability, with a particular focus on how such interventions might influence macrophage phenotype and function. Indeed, in regenerative species, boosting glycolysis while reducing mitochondrial oxidation during the initial phases of regeneration is crucial for driving blastema formation and regeneration 27. Conversely, glycolysis inhibition leads to complete suppression of blastema formation 27. In agreement, promoting glycolysis with oligomycin in SAZ during a specific time window in the early stage of regeneration (short treatment) restored their regeneration capacity. Conversely, oligomycin-induced glycolysis throughout the whole regeneration process resulted in impaired caudal fin regeneration in both control and senescent zebrafish. These results indicate that glycolysis is essential during the early phase of regeneration when macrophages, which require glycolysis for their survival and polarization toward an inflammatory phenotype (*tnfa*^+^ macrophages) 61, 62, mainly accumulate at the wound (with a peak at 6 hpA) and then disappear at a later stage (from 48 to 72 hpA) 22.

As exposure to oligomycin throughout the whole regeneration process affected the regeneration potential of both control and senescent zebrafish, we hypothesize that oligomycin induces prolonged activation of macrophages, thereby altering the rapid resolution of inflammation during regeneration and also fin regeneration. This hypothesis is supported by our previous results showing that during caudal fin regeneration, the finely regulated balance of macrophage subsets provides the precise TNFα signal required to initiate blastemal cell proliferation 24. Moreover, we previously reported that injection of recombinant zebrafish *Tnfa* reduces fin fold regeneration, indicating that a maintained presence of pro-inflammatory cytokines, such as TNFa, impairs regeneration 38. Here, we found that prolonged exposure of zebrafish larvae to oligomycin, which primes macrophages toward a pro-inflammatory phenotype 63, altered the regeneration process. Conversely, the early and transient treatment with oligomycin induced a controlled activation of macrophages to a pro-inflammatory phenotype at the wound site during the first hours of regeneration and regeneration was normalized in SAZ. Surprisingly, the early and transient treatment of control zebrafish with oligomycin significantly reduced their caudal fin regeneration capacity. This effect was associated with a significant lower number of *tnfa*+ macrophages between 6 and 24 hpA, when this subset is necessary for regeneration 22. This could be due to the fact that macrophage activation with oligomycin prior to amputation leads to an early increase in the proportion of pro-inflammatory macrophages under physiological conditions. As a result, *tnfa*+ macrophages accumulate more rapidly at the injury site within the first 3 hours post-amputation, but do not reach a peak between 6 and 24 hpA when TNFR expression, which depends on TNFα produced by macrophages, is required for cell proliferation and blastema formation 22. This hypothesis is supported by the results obtained in *terfa* morphants in which exposure to oligomycin significantly increased the number of *tnfa*+ macrophages at 6 hpA and rescued their regeneration potential. Our results are in accordance with the fact that pro-inflammatory macrophages rely on glycolysis and that glycolysis inhibition in pro-inflammatory macrophages alters their functions, including their phagocytic activity and their capacity to produce reactive oxygen species and to secrete cytokines (for review see 64). Thus, a short oligomycin treatment in the early phases of regeneration could correct the altered glycolytic metabolism in *terfa*-deficient macrophages recruited early after caudal fin amputation. Oligomycin increased the number of *tnfa*+ macrophages recruited at the wound site in SAZ at 6 and 24 hpA to the levels observed in control zebrafish larvae at the same time points. Our study reveals that the need of glycolysis during regeneration is partly related to the fine control of the macrophage response required for blastema formation and regeneration. Further studies are needed to determine whether glycolysis is essential exclusively for the regenerative functions of macrophages or also plays a role in those of other cell types.

In summary, our study elucidates how senescence affects the regenerative capabilities of organisms by profoundly altering the critical functions of macrophages in response to tissue injury. Our discovery highlights the significant role of metabolic changes in governing the capacity of macrophages to express regenerative factors, thereby influencing blastema formation and thus regeneration. This contributes to a deeper understanding of the mechanisms underlying regeneration changes in aging tissues.

## Materials and Methods

### Ethics

Animal experimentation procedures were carried out according to the European Union guidelines for handling of laboratory animals and were approved by the Direction Sanitaire de l'Hérault and Comité d'Ethique pour l'Expérimentation Animale n°036 and APAFIS #32511-2021072114172657 v2.

### Zebrafish lines, maintenance and handling

Fish and embryo maintenance, staging and husbandry were done at the fish facility of the University of Montpellier, France. Experiments were done using wild type (WT) individuals from the AB background and the following transgenic lines: (i) *Tg(mpeg1:eGFP*) to visualize macrophages in green, (ii) *Tg(mpeg1:mCherry*) to visualize macrophages in red, (iii) *Tg(mpeg1:mCherry-F;tnfa:eGFP-F)* to visualize *tnfa* expression (green) in macrophages (red), and (iiii) *Tg(mpeg1:Gal4/UAS:NTR1.0mcherry)* for macrophage depletion with metronidazole treatment. Embryos were obtained from adult fish pairs by natural spawning and raised at 28.5 °C in tank water. Embryos and larvae were used until 6 days post-fertilization (dpf).

### Morpholino injection

*Terfa* was knocked down in zebrafish using a morpholino antisense oligonucleotide (Gene Tools) against the ATG site (*terfa* MO*)*: 5' GGTTCGCAGGGTTTGTCGCTCATTC 3'. A control morpholino (*Ctrl* MO) (Gene Tools) was also used: 5' AATCACAAGCAGTGCAAGCATGATG 3'. Each morpholino was used at a concentration of 100 µM and 2 nL were injected in one-cell stage embryos with a Femto microinjector (Eppendorf).

### CRISPR injection

*Terfa* was silenced in zebrafish using the CRISPR-Cas system as previously described 39. Briefly, three guide RNAs targeting distinct exons were designed on https://chopchop.cbu.uib.no/: gRNA1 (CGTAGAAATCGAAGCTCCAG), gRNA2 (ATCAACAACGGGGACAAGCT), and gRNA3 (ACGCCACGCCATCAGCGAGA). Scrambled sequences were used as control. A mix of the three gRNAs (20 pMol/µL each) with True Cut Cas9 protein v2 (Invitrogen, A36496, 500 ng/µL) was injected in one-cell zygotes (1 nL per egg) with a Femto microinjector (Eppendorf).

### Western blotting

Protein lysates were obtained from 25 zebrafish larvae crushed in 100 µL RIPA buffer supplemented with a protease inhibitor cocktail. After protein quantification with the Micro BCA Protein Assay Kit (ThermoFisher) according to the manufacturer's instructions, 20 μg of proteins per sample were separated by SDS-Page in Laemmli buffer and transferred to nitrocellulose membranes using the iBlot™ 2 Dry Blotting System from Invitrogen. Membranes were blocked with 5% bovine serum albumin (BSA) in Tris-buffered saline with 0.1% Tween-20 (TBST) and incubated with primary antibodies against rabbit TERFA (1/2000; Novus Biologicals, NB110-57130) and mouse actin (1/2500; Sigma, A5441) at 4 °C overnight. Then, membranes were washed with TBST, incubated with secondary antibodies for 1 hour, and finally with the HRP substrate before imaging with the ChemiDoc MP imaging system (BioRad). Quantification was performed using the Image J software (Gel-plot lane function was used).

### Caudal fin amputation

In 3 dpf zebrafish larvae, the caudal fin was amputated with a sterile scalpel, posterior to the muscle and notochord under anesthesia with 160 mg/mL (0.016%) buffered tricaine solution (tricaine, ethyl 3-aminobenzoate; Sigma-Aldrich, France). After amputation, larvae were placed in tricaine-free water at 28.5°C until the study end.

### Oligomycin treatment

Zebrafish larvae were treated with 150 nM oligomycin (Calbiochem) added to the tank water from 24 hours before to 6 hours after amputation (6 hpA) for the short exposure. For the long exposure, oligomycin was added to the zebrafish water from the caudal fin amputation until 72 hpA. After treatment, larvae were rinsed three times in zebrafish water and incubated in tank water until the end of the study.

### Galloflavin treatment

Galloflavin (Tocris Bioscience) was prepared as a 5 mM stock solution in 100% DMSO. For treatments, a 500 nM working solution was obtained by direct dilution in zebrafish water immediately after amputation and maintained until 24 hpA.

### Cell proliferation

Proliferative cells were labeled using an anti-phosphorylated histone 3 (S10) antibody (pH3, Cell Signaling). At each time point, larvae were fixed in 4% paraformaldehyde at 4°C overnight. Then, larvae were rinsed in PBS 0.1% Tween 20, permeabilized with acetone at -20°C for 7 minutes, processed for blocking with the blocking solution, and incubated in blocking solution containing the anti-pH3 antibody (1:500) at 4°C overnight. After washing at room temperature for 10 minutes, larvae were incubated in blocking solution containing the secondary goat anti-Rabbit IgG Alexa Fluor Plus 488 antibody (1:500; ThermoFisher) for 1 hour. Larvae were washed at room temperature in PBS 0.1% Tween 20 before imaging.

### ɣH2AX expression

ɣH2AX-positive cells were detected using an anti-histone H2A-XS139ph (phosphorylated on Ser139) antibody (GeneTex, GTX127342) diluted at 1:500. The secondary antibody was a goat anti-rabbit IgG Alexa Fluor Plus 594 (Thermofisher) diluted at 1:500.

### Imaging and quantification

For imaging, larvae were anesthetized in 0.016% tricaine, immobilized in 35 mm glass bottom dishes (FluoroDish™, World Precision Instruments) using 1% melting point agarose (Sigma-Aldrich) and covered with a small volume of fish water containing 0.016% tricaine to prevent drying out. Confocal imaging of live anesthetized larvae (for the regeneration analysis and to study macrophage recruitment and phagocytic activity) and of fixed larvae (immunofluorescence studies) was done by acquisition of Z-stacks series with inverted confocal microscopes (Leica TCS SP5 and Leica TCS SP8 with Leica Application Suite V3.2 and V3.5, respectively) at the IRMB Cartigen facility. 3D images and video showing debris phagocytosis by macrophages were obtained using an inverted confocal Leica Stellaris 5 microscope and the Aivia software. Images were analyzed using the Fiji-ImageJ software. The caudal fin fold length during regeneration was calculated as the distance between the amputation plane and the edge of the fin fold in the medial plane. The regenerative fin area was calculated as the area of the fin fold from the amputation plane and the fin fold edge. Recruited and activated macrophages, γH2AX-positive cells, and pH3-positive cells were counted directly on microscopy images using the Fiji-ImageJ software.

### Cell suspension preparation and macrophages sorting

For each condition, cells from 50 *Tg*(*mpeg1:mCherry*) larvae were dissociated at 72 hpf. Briefly, the pool of larvae was immersed in 500 µL of FACS MaxTM Cell Dissociation Solution and incubated at room temperature for 5 minutes. Subsequently, tissue dissociation was facilitated by pipetting up and down for 10 minutes. Cell suspensions were then filtered through a 40 µm cell strainer. The cell strainer was rinsed with an additional 1 mL of the FACS MaxTM Cell Dissociation Solution. Then, 1.5 mL of each cell suspension was centrifugated for 5 minutes. Cell pellets were rinsed twice with PBS. Finally, pellets were gently resuspended in DPBS supplemented with 0.4% BSA and filtered one last time through a 40µm cell strainer. Cell sorting was carried out with a Cytek Aurora Cell Sorter (Cytek Biosciences). Settings to ensure gating only of *mCherry*+ cells were used to separate *mCherry*+ macrophages from the other cells. Sorted cells were collected in microfuge tubes containing DPBS 0.4% BSA that were centrifuged for 10 minutes. Cell pellets were resuspended in buffer QIAZOL (miRNeasy Micro Mit, Qiagen) for lysing before RNA isolation.

### Quantitative RT-PCR

Total RNA was isolated from zebrafish caudal fins (pool of 20 fins per condition) using the RNeasy Micro Kit (Qiagen).

Total RNA was isolated from sorted cells using the miRNeasy Micro Kit (Qiagen). RNA concentration and quality were evaluated with a NanoDropOneC spectrophotometer. 200 ng (100 ng from sorted cells) of total RNA was reverse transcribed into cDNA using the M-MLV reverse transcriptase kit (Bioline) according to the manufacturer's instructions. Real-time qPCR assays were performed using the SensiFAST™ SYBR® No-ROX Kit (Bioline, Meridian Life Science Company) and the ViiA 7 Real-Time PCR System. Amplification conditions were: denaturation (1 cycle: 95°C for 10 min), amplification (40 cycles: denaturation 95°C for 10 s, annealing 64°C for 10 s, elongation 72°C for 20 s), and melting curve (95°C for 15 s, 65°C for 1 min, 95°C for 15s). The primer sequences (SYBR Green Technology) were: *cdkn2a/b* (5'-CGAGGATGAACTGACCACAGC-3'), *cdkn2a/b* (5'-CAAGAGCCAAAGGTGCGTTAC-3'), *cdkn1a* (5'-CCGCATGAAGTGGAGAAAAC-3'), *cdkn1a* (5'-ACGCTTCTTGGCTTGGTAGA-3'), *tp53* (5'-GCGATCATGGATTTAGGCTC-3'), *tp53* (5'-CTTATAGATGGCAGTGGCTCG-3'), *lin28* (5'-TAACGTGCGGATGGGCTTCGGATTTCTGTC-3'), *lin28* (5'-ATTGGGTCCTCCACAGTTGAAGCATCGATC-3'), *il-6* (5'-TGAAGACACTCAGAGACGAGCAGTT-3'), *il-6* (5'-AGGTTTGAGGAGAGGAGTGCTGAT-3'), *mmp9* (5'-CTCAGAGAGACAGTTCTGGG-3') and *mmp9* (5'-CCTTTACATCAAGTCTCCAG-3'), *ldha* (5'-GAGCGGTTTGCCCAGGAACC-3'), *ldha* (5'-GAGGGACACCCCCACAT-3'), *eno3* (5'-GTTTGCAGGAAAAGACTTCCG-3'), *eno3* (5'-CAACTCTTCAATGCACGCTT-3'), *pdhb* (5'-GTTCGAGATGTCGGAAGAGG-3') and *pdhb* (GCCTGATAGAACGCAGGTTT-3'). Results were normalized to the housekeeping gene *ef1a*, amplified with the following primers: ef1a (5′-TTCTGTTACCTGGCAAAGGG-3′) and ef1a (5′-TTCAGTTTGTCCAACACCCA-3′).

For experiments on cell in culture, RT-qPCR was performed with the same protocol. Primers sequence to analyze *terfa* expression were: *terfa* (GCA GAA GAT GCT GCG TTT CCT AG) and *terfa* (TTT CCA CTG GCT CTG GGT GCT T). Results were normalized to the housekeeping gene *18S*: *18S* (GCC CGA AGC GTT TAC TTT GA) and *18S* (TTG CGC CGG TCC AAG AAT TT).

### Locomotion test

Motility was monitored in 120 hpf zebrafish larvae using the Zebrabox recording system (Viewpoint). Motility was recorded in the dark for 5 minutes after one cycle of 5 minutes of light excitation. Slow velocity (3-6 mm / s), high velocity (> 6 mm / s) and inactive time were recorded to determine the net velocity and the duration of the motility period during the assay, respectively.

### Parabiosis

The *terfa* MO was injected in the zebrafish transgenic line *Tg(mpeg1:Gal4/UAS: NTR1.0mcherry)* at the one-cell stage. WT embryos and transgenic embryos were dechorionated at the 256-cell stage in glass Petri dishes with E3 medium (5 mM NaCl, 0.17mM KCl, 0.33 mM CaCl_2_, 0.33 mM MgSO_4_) containing antibiotics (50 U/mL penicillin-streptomycin, 1 mM ampicillin). Two epiboly stage embryos (one for each zebrafish line) were transferred into a hand-made agarose mold that contained small wells filled with high-calcium Ringer's solution (5 mM NaCl, 0.17 mM KCl, 5 mM CaCl_2_, 0.33 mM MgSO_4_). Few cells were detached from each embryo using a glass capillary near their contact point. Then, embryos were rapidly pushed to press the wounds against each other in order to allow their fusion. After fusion, embryos were incubated at 28.5°C for 1 hour. Then, the high-calcium Ringer's solution was replaced by E3 medium and the fused embryos were kept at 28.5°C overnight. The E3 medium was slowly replaced by tank water at 24 hpf. At 48 hpf, macrophages from *Tg(mpeg1:Gal4/UAS:NTR1.0mcherry)* larvae were depleted by adding 10 mM metronidazole (Fisher Scientific) in zebrafish water until the end of the parabiosis experiment.

### Labeled cell debris injection in zebrafish embryos

Labeled cell debris was prepared as described in the Phagocytosis assay section. 3 hours before caudal fin injury, 2 nL of fluorescent cell debris was injected into the caudal vein of embryos anesthetized with 0.016 % tricaine. After injection, larvae were placed in tank water until the experiment end.

### Liposome preparation and injection

Liposomes were synthesized using the thin lipid film hydration method and had the following composition: phosphatidyl choline (PC): cholesterol: 95:5 (mol:mol). Briefly, PC and cholesterol were weighed and dissolved in chloroform to 5 mg/mL total lipid concentration. The fluorescent probe 1,1′-Dioctadecyl-3,3,3′,3′-Tetramethylindocarbocyanine perchlorate (DiI) was added to the lipid solution to obtain a 3 µM final concentration in the suspension. Chloroform was evaporated using a rotary evaporator and the dried film was resuspended in DPBS at 3 mg/mL and stored for hydration at 4°C overnight. The hydrated liposome suspension was then extruded using a pressure triggered extrusion system (LIPEX^®^ 10mL Thermobarrel Extruder Package Evonic). Liposomes were then washed three times with PBS by ultracentrifugation (100,000 g, 30 minutes). The liposome hydrodynamic diameter was determined by dynamic light scattering (NanoZS, Malvern Instruments, UK, equipped with a He-Ne laser, wavelength: 632.8 nm), at 25°C and a scattering angle of 173° for detection. Liposomes were stored at 4°C until injection 3 hours before injury. For each injection, 2 nL of DiI-labeled liposomes was microinjected into the tail vein of zebrafish embryos anesthetized with 0.016% tricaine. After injection, larvae were placed in tank water until the experiment end.

### Cell culture

RAW 264.7 murine macrophages were cultured in high-glucose Dulbecco's Modified Eagle Medium (DMEM) (Thermo Fisher Scientific, MA, USA) supplemented with 10% heat-inactivated fetal bovine serum (Biowest, Nuaillé, France), 2 mM L-glutamine and 1% penicillin/streptomycin (Gibco - ThermoFisher Scientific) at 37°C, 5% CO_2_. Cells were used between passages 8 and 12.

### Cell transfection

RAW 264.7 macrophages were seeded in tissue culture-treated 96-well flat bottom plates at the density of 28 000 cells/cm^2^ and transfected overnight with 20 nM of control siRNA or siRNA against *terfa* (silencer pre-designed siRNA, Ambion, Life Technologies™), using Oligofectamine Transfection Reagent (Invitrogen), following the provider's instructions. The sequences of the anti-*terfa* siRNAs were: GCUUUGAAAUCUGAAUCAGtt (sense) and CUGAUUCAGAUUUCAAAGCtt (antisense).

### Phagocytosis assay

Following transfection, RAW 264.7 macrophages were activated with LPS (250 ng/mL) (Invitrogen) and IFN-γ (20 ng/mL) (R&D Systems) in assay medium containing DMEM without phenol red (Thermofisher) supplemented with 10% heat-inactivated fetal bovine serum (Biowest), 25 mM D-glucose, 25 mM HEPES, 1X pyruvate, 2 mM L-glutamine, 1% MEM-Non-essential Amino Acids, 1% penicillin/streptomycin and 50 μM 2-mercaptoethanol (Gibco) for 6 hours. Cell debris were prepared as follows. Murine mesenchymal stromal cells (MSCs) were labeled with DiD (1 μM) (Invitrogen) for 20 minutes. After washing, MSCs were lysed twice with a tissue homogenizer (ULTRA-TURRAX) at speed 6 for 30 seconds. Then, labeled-MSC lysates were centrifuged for 10 minutes and pellets resuspended in assay medium. The equivalent of 10,000 MSCs were added per well. Debris uptake by RAW 264.7 macrophages was followed using a Cytation 5 plate reader (BioTek, Agilent, Winooski, Vermont, U.S.A) and the Gen5 software. A total of four images per well were taken, and images were preprocessed with a background subtraction step before the qualitative analysis.

## Supplementary Material

Supplementary figures.

Supplementary video.

## Abbreviations

SAZ: senescence-accelerated zebrafish
LDHA: lactate dehydrogenase A
WT: wild-type
TNF: tumor necrosis factor
MO: morpholino
dpf: day post-fertilization
hpA: hour post-amputation
CTRL: control
SASP: senescence-associated secretory phenotype
pH3: phosphorylated histone 3
MTZ: metronidazole
ENO3: enolase 3
PDHB: Pyruvate Dehydrogenase beta
LPS: lipopolysaccharide
IFN-g: interferon gamma
