# Supplementary material for: Disrupted macrophage metabolic adaptation and function drive senescence-induced decline in vertebrate regeneration
Source: Theranostics. 2025 Jun 20;15(15):7308–26. doi: 10.7150/thno.111352 (PMC12315812; doi:10.7150/thno.111352)

Supplementary Figure 1

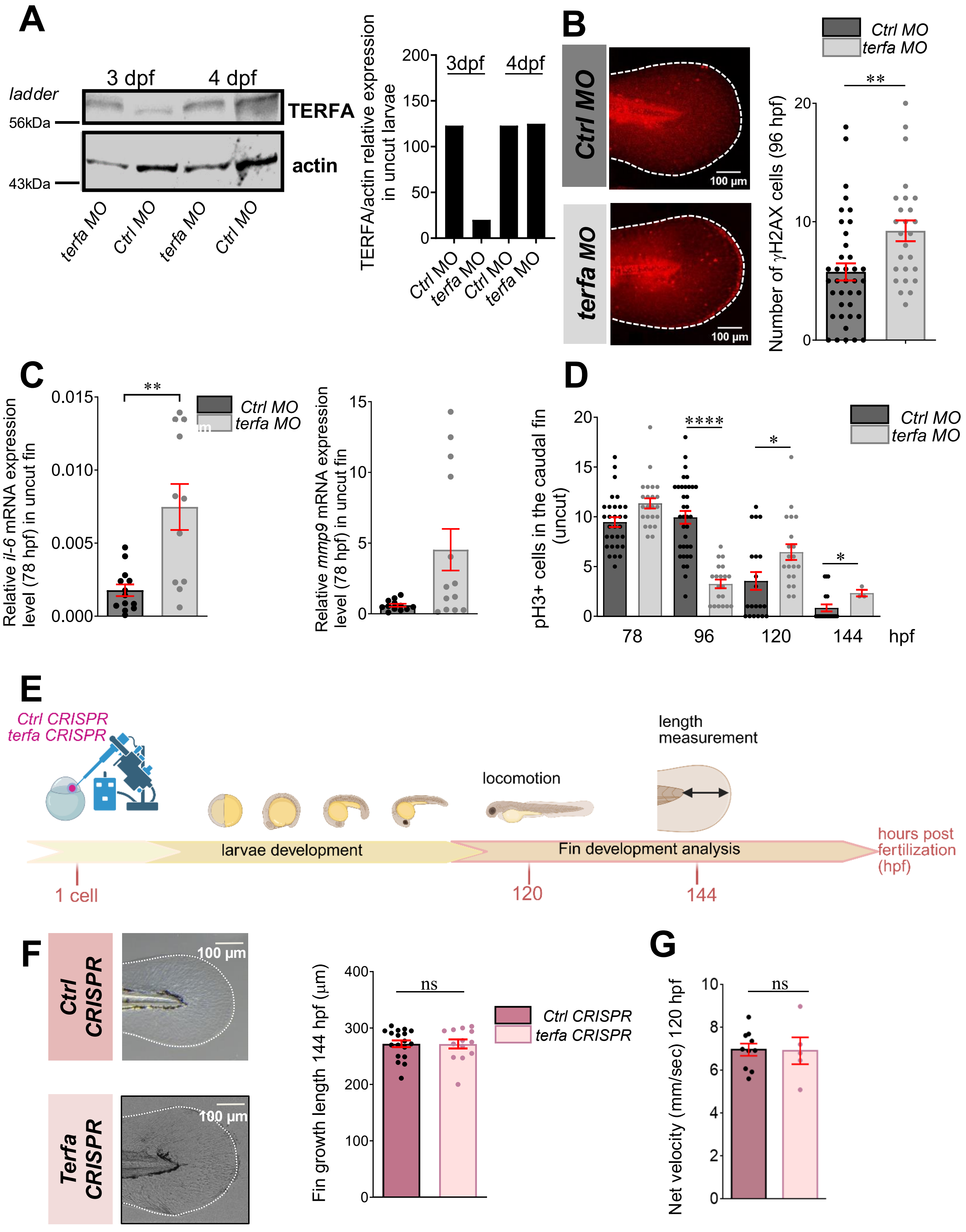

Supplementary Figure 2

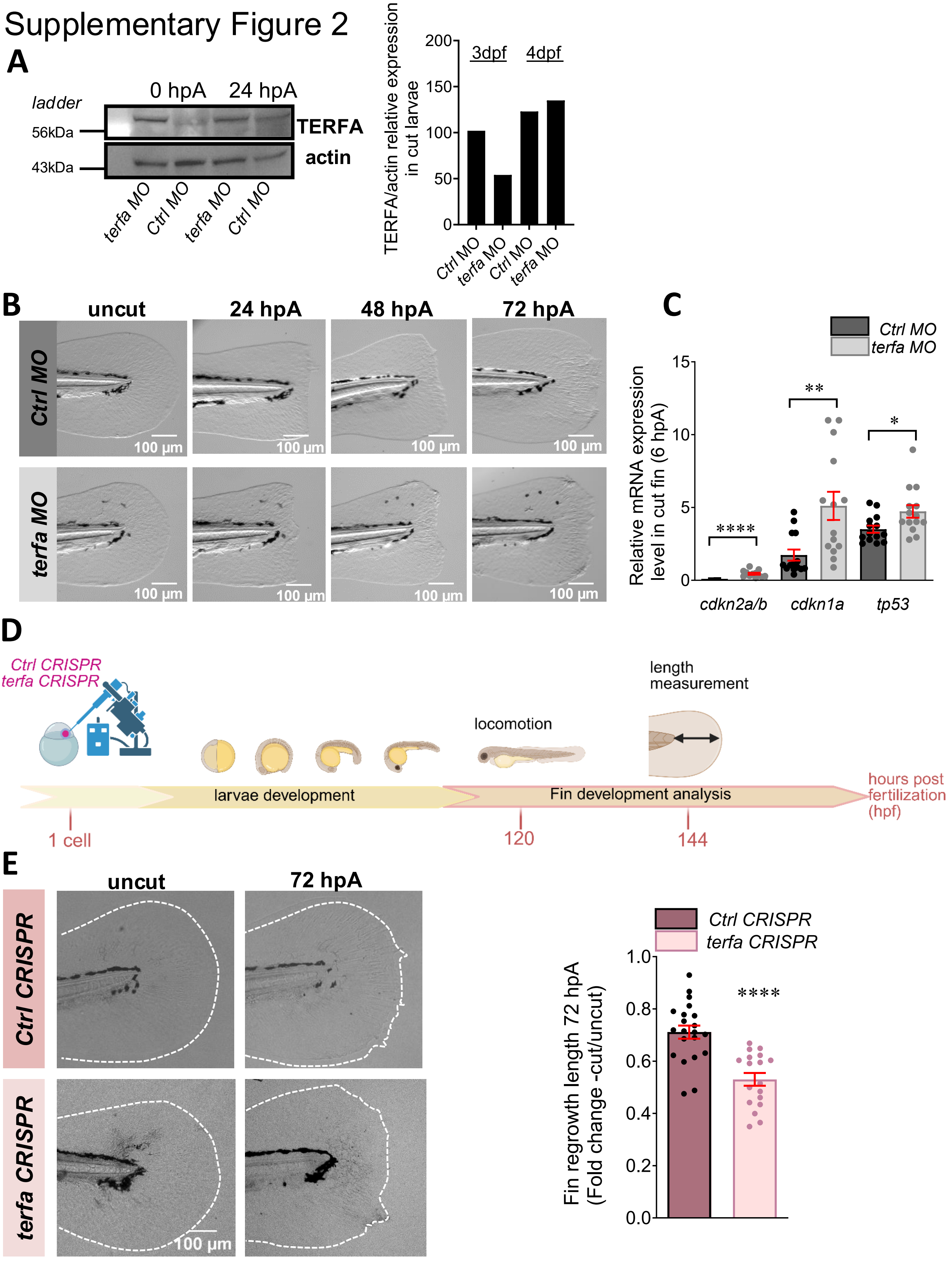

Supplementary Figure 3

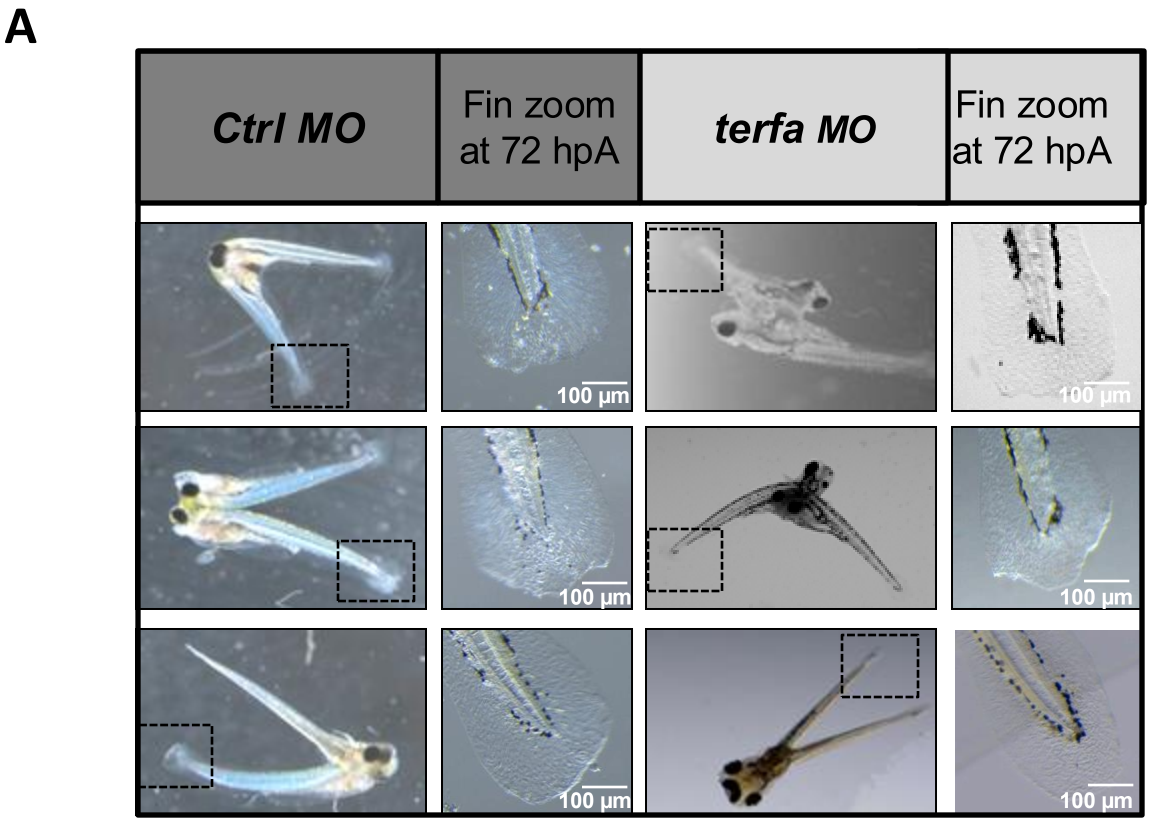

Supplementary Figure 4

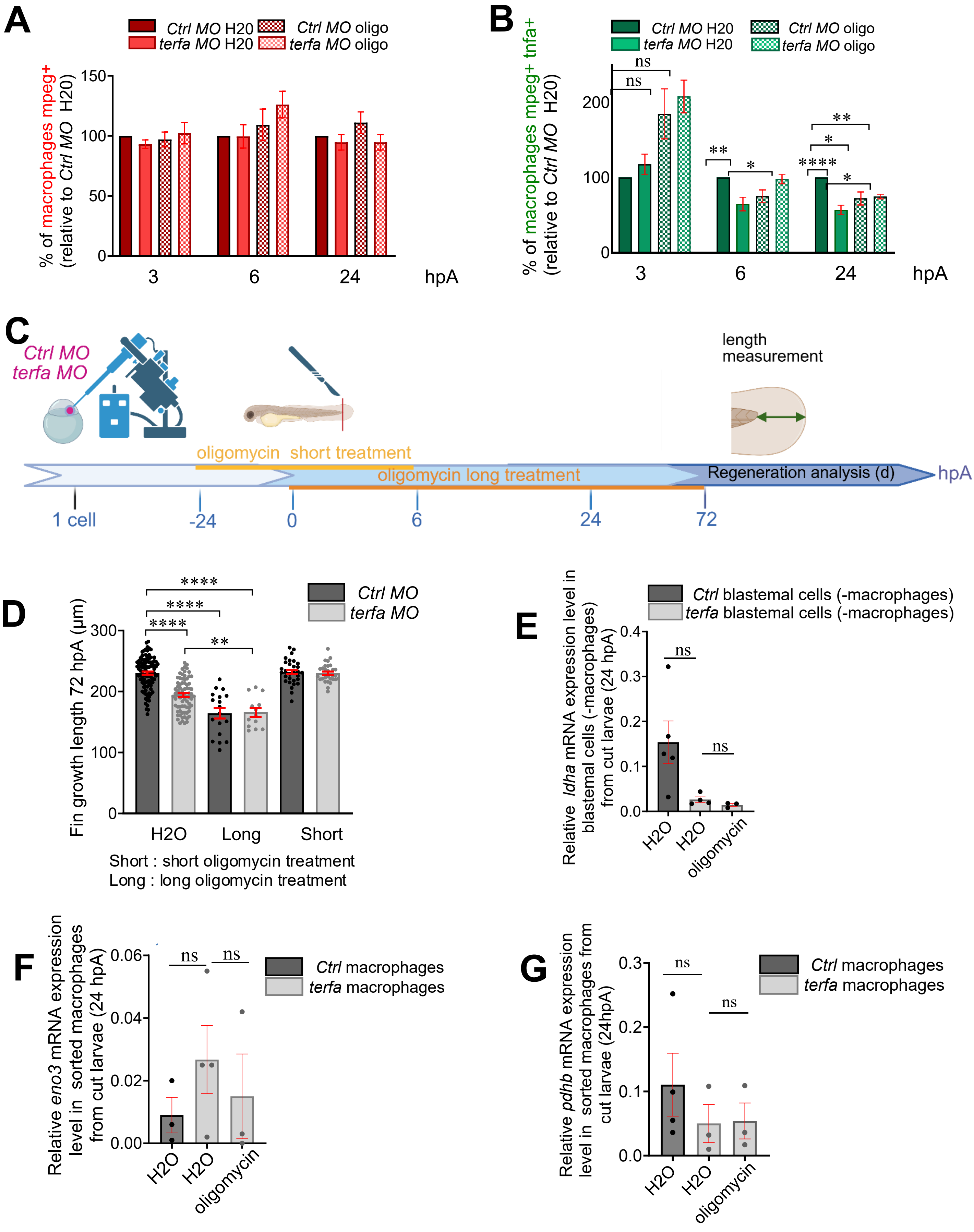

Supplementary Figure 5

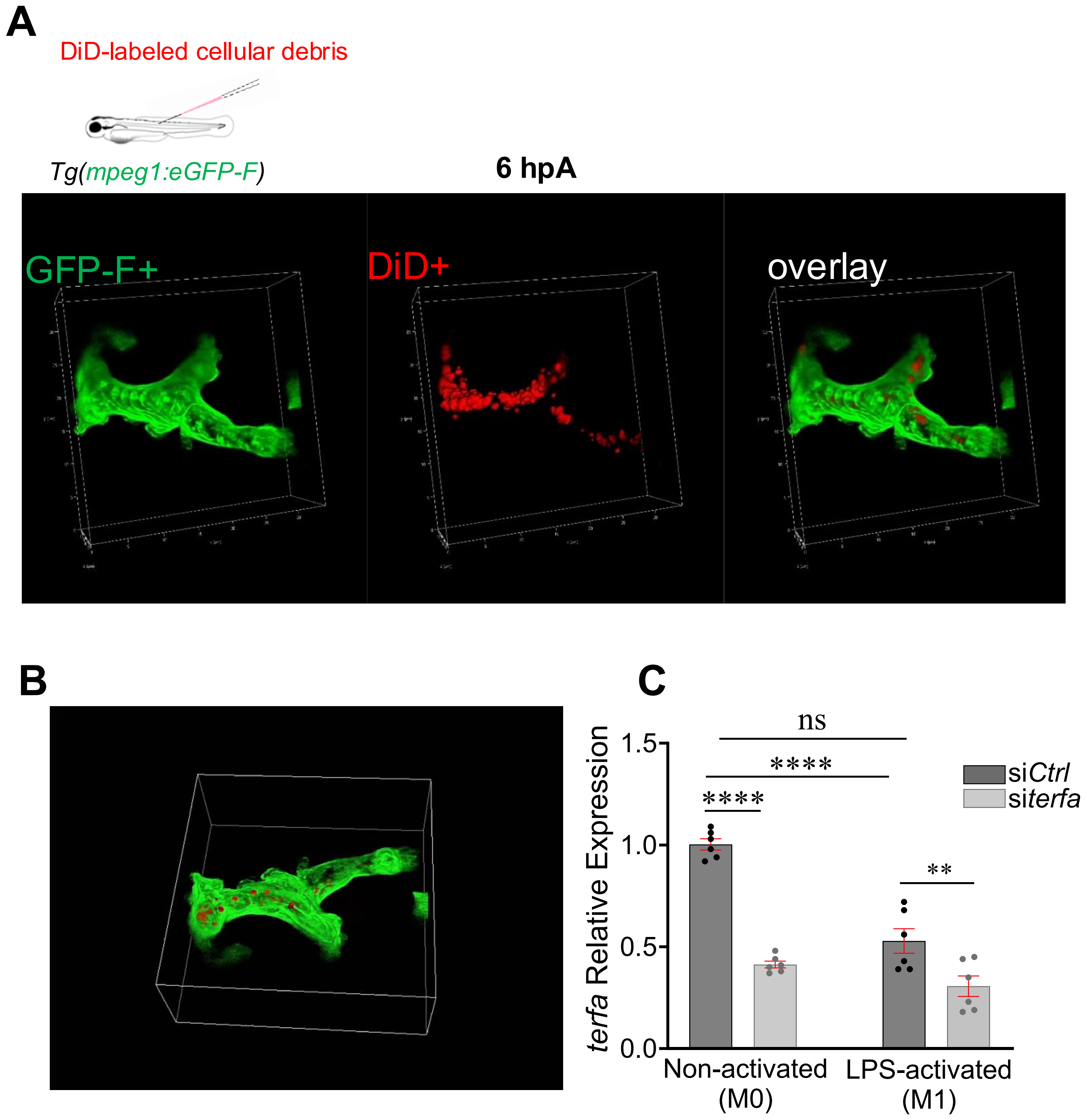

Supplement: Supplementary file 1 — Supplementary figures. [file thnov15p7308s1.pdf]
